# Smoking Is a Risk Factor of Coronary Heart Disease through HDL-C in Chinese T2DM Patients: A Mediation Analysis

**DOI:** 10.1155/2020/8876812

**Published:** 2020-07-28

**Authors:** Ru Tang, Shanshan Yang, Weiguo Liu, Bo Yang, Shuang Wang, Zhengguo Yang, Yao He

**Affiliations:** ^1^The 2^nd^ Medical Center, Chinese PLA General Hospital, Beijing 100853, China; ^2^Department of Disease Control and Prevention, The 1st Medical Center, Chinese PLA General Hospital, Beijing 100853, China; ^3^Institute of Geriatrics, Beijing Key Laboratory of Aging and Geriatrics, National Clinical Research Center for Geriatrics Disease, State Key Laboratory of Kidney Disease, The 2^nd^ Medical Center, Chinese PLA General Hospital, Beijing 100853, China; ^4^Emergency Department, Armed Police Corps Hospital in Henan Province, Zhengzhou 450000, China; ^5^Department of Nephrology and Endocrinology, PLA 960^th^ Hospital, Zibo 255300, China

## Abstract

**Objective:**

To investigate associations between smoking and cardiovascular and cerebrovascular complications in type 2 diabetes mellitus (T2DM) patients.

**Methods:**

This is a cross-sectional study. Of 971 T2DM patients aged 14–93 years old in this study, 182 had ever smoked and 789 never smoked. Propensity score matching (PSM) reduced the confounding bias between groups. Logistic regression analysis was performed on matched data to evaluate coronary heart disease (CHD) and stroke risk. In addition, the mediation analysis was conducted among smoking exposure, HDL-C, and CHD.

**Results:**

A total of 139 pairs of patients who had never and ever smoked were matched. Logistic regression analysis showed that compared with patients who never smoked, those who smoked > 20 cigarettes per day (CPD) had a higher risk of CHD (odds ratio [OR]: 3.09, 95% confidence interval [CI]: 1.21–7.89). Additionally, after adjusting for age, sex, origin, occupation, smoking status, body mass index, waist circumference, and diabetes duration, the OR for CHD with >20 years of cumulative smoking (pack-years) was 2.21 (95% CI: 1.05–4.65). Furthermore, we observed a significant dose-response relationship between CPD and lower high-density lipoprotein cholesterol (HDL-C) (*P* < 0.001). Moreover, the mediation analysis showed that the indirect effect mediated by HDL-C accounted for 86% (effect = 0.0187, 95% CI: 0.0100–0.0316).

**Conclusions:**

Smoking may be a risk factor for CHD in T2DM patients. T2DM patients should stop smoking or reduce the CPD to prevent the onset of CHD. Moreover, to prevent CHD complications, monitoring HDL-C levels in T2DM patients who smoke may be necessary.

## 1. Introduction

More than 5% of adults worldwide have type 2 diabetes mellitus (T2DM), and the prevalence will increase to 6.3% by 2025 [1]. In China, an estimated 23.46 million people currently have diabetes, and that number is predicted to increase to 42.30 million by 2030 [2, 3]. Coronary heart disease (CHD) and stroke are the most common chronic complications of T2DM and the main cause of T2DM-related mortality [4]. Patients with T2DM have a 1.54–4.00 times higher risk of CHD [5, 6] and 1.35–1.74-times higher risk of stroke [7]. In China, the annual per patient cost of healthcare associated with T2DM patients with cardiovascular and cerebrovascular complications is estimated to be 1798 USD, compared with 484 USD for those without these complications [2]. Moreover, previous studies also showed that the morbidity of cerebrovascular disease is 2–5 times higher in patients with T2DM than in patients without T2DM [8]. Thus, identifying risk factors, especially preventable risk factors, for cardiovascular and cerebrovascular complications in T2DM is important.

Cigarette smoking is an important modifiable risk factor for cardiovascular and cerebrovascular disease in a general population [9, 10]. However, this relationship is less well-defined among individuals with diabetes [11], especially in Chinese diabetic inpatients. In previous studies, smoking exposure was usually categorized as never, current, and past only, but objective data of smoking exposure such as cigarettes per day (CPD), time of smoking, and cumulative smoking (pack-years) was not provided in these studies [12, 13]. Furthermore, the combined effect of smoking and the influence of blood pressure, glucose, and serum lipids on cardiovascular and cerebrovascular disease is also unclear. The mechanism by which smoking affects cardiovascular and cerebrovascular diseases is not clear, either. In addition, in previous studies, demographic characteristics of groups who ever and never smoked differed significantly. Thus, we designed a study to assess the association between smoking and CHD/stroke in Chinese T2DM patients using CPD, time of smoking, and cumulative smoking (pack-years) to measure smoking exposure, in addition to propensity score matching (PSM) to control for differences in characteristics between those who never and ever smoked. Further, mediation analysis was used to explore the mechanism of smoking exposure on CHD in Chinese T2DM patients.

## 2. Design and Methods

### 2.1. Study Sample

**Figure 1:**
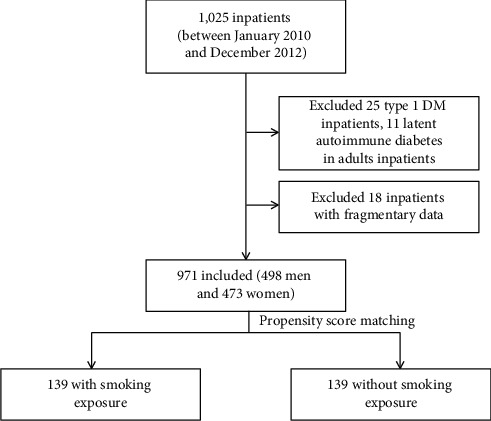
The flowchart of participants.

We used clinical data from the Department of Nephrology and Endocrinology, PLA 148^th^ Hospital (renamed as PLA 960^th^ Hospital now). Among 1,025 inpatients (between January 2010 and December 2012), we excluded 25 type 1 DM inpatients, 11 latent autoimmune diabetes in adults inpatients, and 18 inpatients with fragmentary data and recruited 971 (498 men and 473 women) as our participants (Figure 1).

We collected data regarding each participant's sex, age, occupation, region, alcohol and smoking consumption, diabetes duration, and CHD and stroke status.

### 2.2. Measurements

T2DM was defined according to the American Diabetes Association criteria [14]. CHD and stroke were defined using the WHO MONICA criteria [15] by physicians of the PLA 148^th^ Hospital.

A smoker was defined as a person who had smoked daily for at least 6 months during their lives [16]. CPD, time of smoking, and cumulative smoking (pack-years) were used to measure cigarette consumption. An alcohol user was defined as a regular drinker who consumed alcohol approximately daily and had been regularly consuming alcohol for more than 6 months [3].

The information was collected by a primary nurse. Height was measured in meters (without shoes). Weight was measured in kilograms, without heavy clothing and 1 kg deducted for remaining garments. Body mass index (BMI, kg/m^2^) was calculated as weight in kilograms divided by the square of height in meters. To ensure the accuracy of the information, patient answers to the questions on tobacco use were confirmed by the patients and their relatives. Central obesity was defined by a waist circumference (WC) > 90 cm in men and > 80 cm in women [17]. Venous blood was taken after fasting of eight hours. Fasting blood-glucose (FBG), hemoglobin A1c (HbA1c), cholesterol (CHO), and high-density lipoprotein cholesterol (HDL-C) were tested in the central laboratory of PLA 148th Hospital. Covariables adjusted in the study included age, sex (male and female), occupation (white collar, light physical labor and hard physical labor), region (Shandong province and other provinces), drink (yes and no), BMI, WC, and diabetes duration.

### 2.3. Statistical Analysis

SPSS version 19.0 was used for data analysis. The significance level for all tests was set at a two-tailed *α* value of 0.05. The differences in means and proportions were evaluated using Student's *t*-test and the chi-square test, respectively. Logistic regression models were used to identify the risk of tobacco use and linear regression models were used to identify the effect of tobacco use on FBG, HbA1c, CHO, HDL-C, and systolic pressure.

PSM [18] was used to match groups of those who did and did not consume tobacco. Sex, age, origin, occupation, drinking status, BMI, WC, and T2DM duration were included as covariates. We used nearest-neighbor matching to pair never smokers with current and former smokers at a 1 : 1 ratio with a caliper width of 0.02 [19].

Mediation analysis is a method which is used to assess the relative magnitude of different pathways and mechanisms by which an exposure may affect an outcome [20]. Mediation analysis was used to analyze the indirect effect on CPD and CHD mediated by HDL-C. Extended program of SPSS was used to do the mediation analysis (model 4 [21] was used to simulate the mediation effect, and the conceptual diagram is shown in Figure S1).

### 2.4. Ethical Considerations

The committee for medical ethics of the Chinese PLA General Hospital examined and approved our study. Before completing the questionnaire, each involved participant signed an informed consent form.

## 3. Results

A total of 971 (498 men and 473 women) inpatients were involved in our study before PSM. The average age was 56.8 ± 11.6 years (range: 14–93 years). The average ages of those who did and did not use tobacco were 53.5 ± 11.9 years and 57.6 ± 11.4 years, respectively. The general characteristics (age, sex, origin, occupation, drinking status, BMI, and central obesity) of the participants are shown in Table 1. Compared with the group of ever smokers, the group who never smoked consisted of more women, more hard physical laborers, fewer drinkers, and patients who were older and had less central obesity and longer T2DM durations (6.3 ± 6.1 years vs. 7.4 ± 6.6 years; *P*=0.05).

After PSM, a total of 139 participant pairs were matched, and the two groups were balanced for age, sex, occupation, drinking status, BMI, central obesity, and T2DM duration (ever and never smokers: 6.8 ± 6.3 years vs. 6.6 ± 6.3 years, respectively; *P*=0.849) (Table 1).

In logistic regression, we found that compared with never smokers, ever smokers with CPD > 20/day had a higher risk of CHD (OR: 3.09, 95% CI: 1.21–7.89) after adjusting for age, sex, origin, occupation, drinking, BMI, WC, and diabetes duration. We also observed a dose-response relationship between CPD and CHD risk (after adjustment, *P*=0.021). Similar results were observed in cumulative smoking (pack-years) (Table 2). In addition, compared with never smokers, ever smokers with smoking years >20 years had a higher risk of CHD in a crude model (OR: 2.06, 95% CI: 1.08–3.95), and a dose-response relationship between years of tobacco use and risk of CHD was also observed in a crude model (*P*=0.027); however, after adjustment, the effect was no longer significant (Table 2). We also examined the effect of ever smoking on stroke risk, and no significant effect was observed (Table 3).

Further, we examined the effect of CPD, smoking years, and cumulative smoking (pack-years) on FBG, HbA1c, CHO, HDL-C, and systolic pressure, and the results are shown in Table 4. CPD was negatively correlated with HDL-C in T2DM inpatients after adjustment (*β* = −0.006, standard *β*= −0.312, *P* < 0.001).

Further, the mediation analysis showed that the indirect effect on CPD and CHD mediated by HDL-C accounted for 86% (effect = 0.0187, 95% CI: 0.0100–0.0316), and the direct effect of CPD on CHD was 0.0030 (95% CI: −0.0183–0.0242).

## 4. Discussion

In this study, we observed a significant association between smoking exposure and CHD in T2DM patients; however, the association between smoking exposure and stroke was not significant in this population. We used PSM to comprehensively control and adjust for a wide range of potential confounders and to improve the comparability between the two groups (never and ever smokers). Further, we observed a dose-response relationship between CPD and cumulative smoking (pack-years) and the risk of CHD; this relationship was also observed between CPD and lower HDL-C in these T2DM patients.

A study of a Middle Eastern cohort [13] showed that, in men with diabetes, the HR (95% CI) of comparing current smokers and nonsmokers was 1.25 (0.74–2.12) for incident CHD, while, among nondiabetic men, current smokers showed significant risk for CHD (HR = 1.49, 1.18–1.89); however, the study did not assess the association between CPD and the risk of CHD. Another study in the US population (the National Health Interview Survey) [12] also showed that the OR of current smoking for CHD in T2DM patients was 1.61 (95% CI: 0.98–2.65) after adjustment; however, the study only used current, former, and never smoking as the measurement of exposure and did not provide information of CPD, smoking time, or cumulative smoking (pack-years). While the Nurses' Health Study in the US female population [11] showed that, in T2DM patients, compared with never smokers, the risk ratios for CHD were 1.66 (95% CI, 1.10–2.52) for current smokers of 1 to 14 CPD and 2.68 (95% CI, 2.07–3.48) for current smokers of 15 or more CPD after adjustment, our results were similar.

A study of 1,836 Chinese [22] found that participants with both diabetes and a smoking habit had an 8.94-times (95% CI: 3.77–21.19) higher risk of stroke compared with those without diabetes and a smoking habit; however, this study did not provide a comparison between T2DM patients with and without smoking exposure. Another study in a Swedish population involving 13,087 patients with T2DM [23] found that the adjusted HR of smoking for stroke was 1.3 (95% CI: 1.1–1.6); however, the study did not show the relationship between CPD and stroke in T2DM patients. We did not observe a significant association between smoking exposure and stroke in our study, possibly due to the limited sample size.

We also observed a dose-response relationship between CPD and lower HDL-C. In addition, the mediation analysis showed that the indirect effect mediated by HDL-C accounted for 86% on the association between smoking exposure and CHD in the T2DM inpatients. Previous studies showed that HDL-C can exhibit anti-inflammatory properties [24]. Moreover, HDL-C from T2DM patients with CHD stimulated the release of tumor necrosis factor-*α* (TNF-*α*) in monocytes to a greater extent than that of HDL-C from those without CHD, and HDL-C was a significant predictor of the presence of CHD in patients with T2DM [25]. This may indicate that smoking exposure increases the risk of CHD by reducing the level of HDL-C in T2DM patients.

This study had several limitations. As the information on smoking exposure was based on recall, bias could not be fully ruled out; however, the information was confirmed with patients and their relatives to ensure accuracy. Second, our sample may not be completely representative of T2DM patients in China because our hospital is one of the best hospitals in Zibo, and the inpatients here have higher proportions of diabetic complications; however, the representativeness of our sample should not substantially affect the internal validity of this study. Finally, we could not examine the hazard ratio (HR) of smoking exposure with respect to CHD because of the lack of detailed information regarding the onset time of CHD.

In summary, our study found a dose-response relationship between smoking exposure and CHD among T2DM inpatients. We used the PSM method to increase the comparability of the never and ever smokers groups. We also observed a dose-response relationship between CPD and lower HDL-C, which may indicate that smoking exposure increased the risk of CHD by influencing the level of HDL-C in T2DM patients. However, further cohort studies should be conducted to verify the causal relationship. Our findings demonstrated that T2DM patients should be advised to stop smoking, or at least to reduce the amount of CPD, to prevent the onset of CHD. Moreover, to prevent the onset of CHD complications in T2DM patients, monitoring of HDL-C levels in T2DM patients with a smoking habit may be necessary.

## Figures and Tables

**Table 1:** Demographic characteristics according to tobacco use before and after propensity score matching (PSM).

Group	Number (%)	Ever smoking (before PSM)			Ever smoking (after PSM)		
	Total *N* = 971	Yes (*n* = 182)	None (*n* = 789)	*P*	Yes (*n* = 139)	None (*n* = 139)	*P*
Age (years)				0.001			0.728
≤60	81 (8.3)	24 (13.2)	57 (7.2)		15 (10.8)	19 (13.7)	
60–69	529 (54.5)	109 (59.9)	420 (53.2)		85 (61.2)	80 (57.6)	
≥70	361 (37.2)	49 (26.9)	312 (39.5)		39 (28.1)	40 (28.8)	

Sex				<0.001			1.000
Male	498 (51.3)	177 (97.3)	321 (40.7)		136 (97.8)	136 (97.8)	
Female	473 (48.7)	5 (2.7)	468 (59.3)		3 (2.2)	3 (2.2)	

Occupation				0.014			0.464
White collar	103 (10.6)	27 (14.8)	76 (9.6)		21 (15.1)	15 (10.8)	
Light physical labor	117 (12.0)	29 (15.9)	88 (11.2)		21 (15.1)	26 (18.7)	
Hard physical labor	751 (77.3)	126 (69.2)	625 (79.2)		97 (69.8)	98 (70.5)	

Region				0.397			0.562
Shandong province	940 (96.8)	178 (97.8)	862 (96.6)		138 (99.3)	137 (98.6)	
Other provinces	31 (3.2)	4 (2.2)	27 (3.4)		1 (0.7)	2 (1.4)	

Drink				<0.001			1.000
Yes	182 (18.7)	90 (49.5)	48 (6.1)		47 (33.8)	47 (33.8)	
No	789 (81.3)	92 (50.5)	741 (93.9)		92 (66.2)	92 (66.2)	

BMI				0.002			0.167
<24.00	368 (37.9)	39 (21.4)	317 (40.2)		43 (30.9)	46 (33.1)	
24.00–27.99	388 (40.0)	92 (50.5)	296 (37.5)		70 (50.4)	56 (40.3)	
≥28.00	215 (22.1)	51 (28.0)	176 (22.3)		26 (18.7)	37 (26.6)	

Central obesity				<0.001			0.472
Yes	625 (64.4)	90 (49.5)	535 (67.8)		71 (51.1)	65 (46.8)	
No	346 (35.6)	92 (50.5)	254 (32.2)		68 (48.9)	74 (53.2)	

Mean ± SD							
Age	56.8 ± 11.6	53.5 ± 11.9	57.6 ± 11.4	<0.001	54.2 ± 11.7	53.4 ± 13.1	0.583
Duration of diabetes	7.3 ± 6.5	6.3 ± 6.1	7.4 ± 6.6	0.05	6.8 ± 6.3	6.6 ± 6.3	0.849
BMI	25.3 ± 4.1	25.6 ± 3.6	25.3 ± 4.2	0.333	25.4 ± 3.6	25.6 ± 4.0	0.552
WC	88.6 ± 8.8	90.7 ± 8.3	88.2 ± 8.9	0.001	90.7 ± 7.9	90.6 ± 8.6	0.904

**Table 2:** Odds ratio (95% confidence interval, CI) of CHD for smoking in participants.

		*N* (%)	Model A	Model B	Model C
			OR (95% CI)	OR (95% CI)	OR (95% CI)
Smoking					
	None (reference)	29 (20.9)	1	1	1
	Yes	35 (25.2)	1.28 (0.73–2.24)	1.29 (0.69–2.40)	1.36 (0.72–2.58)
	*P*		0.393	0.429	0.347

CPD					
	None (reference)	29 (20.9)	1	1	1
	≤20 (day)	23 (21.7)	1.05 (0.57–1.95)	0.95 (0.48–1.90)	1.00 (0.49–2.04)
	>20 (day)	12 (36.4)	2.17 (0.96–4.92)	**3.00 (1.19–7.55)**	**3.09 (1.21–7.89)**
	*P* for trend		0.127	0.076	0.05

Time of smoking					
	None (reference)	29 (20.9)	1	1	1
	≤20 years	11 (15.7)	0.70 (0.33–1.50)	0.95 (0.41–2.21)	0.97 (0.41–2.29)
	>20 years	23 (35.4)	**2.06 (1.08–3.95)**	1.51 (0.74–3.08)	1.59 (0.77–3.30)
	*P* for trend		0.058	0.294	0.243

Cumulative smoking					
	None (reference)	29 (20.9)	1	1	1
	≤20 pack-years	11 (14.7)	0.65 (0.31–1.39)	0.70 (0.30–1.61)	0.74 (0.31–1.73)
	>20 pack-years	24 (37.5)	**2.28 (1.19–4.36)**	**2.08 (1.01–4.28)**	**2.21 (1.05–4.65)**
	*P* for trend		0.032	0.060	0.026

Variables are included as continuous variables					
	None (reference)		1	1	1
	CPD		**1.02 (1.00–1.04)**	**1.03 (1.00–1.05)**	**1.03 (1.00–1.05)**
	*P*		0.055	0.024	0.021
	None (reference)		1	1	1
	Smoking time (years)		**1.02 (1.00–1.04)**	1.00 (0.99–1.03)	1.01 (0.99–1.03)
	*P*		0.027	0.36	0.251
	None (reference)		1	1	1
	Cumulative smoking (pack-years)		**1.02 (1.01–1.03)**	**1.02 (1.00–1.03)**	**1.02 (1.00–1.03)**
	*P*		0.008	0.037	0.032

Model A: crude model; Model B: adjusted for age, sex, origin, and occupation; Model C: adjusted for age, sex, origin, occupation, drinking, BMI, WC, and diabetes duration; and CPD: cigarettes per day.

**Table 3:** Odds ratio (95% confidence interval, CI) of stroke for smoking in participants.

		*N* (%)	Model A	Model B	Model C
			OR (95% CI)	OR (95% CI)	OR (95% CI)
Smoking					
	None (reference)	18 (12.9)	1	1	1
	Yes	21 (15.1)	1.20 (0.61–2.36)	1.15 (0.55–2.53)	1.25 (0.57–2.74)
	*P*		0.605	0.673	0.573

CPD					
	None (reference)	18 (12.9)	1	1	1
	≤20 (day)	14 (13.2)	1.02 (0.48–2.16)	0.90 (0.38–2.09)	0.94 (0.40–2.25)
	>20 (day)	7 (21.2)	1.81 (0.69–4.78)	2.68 (0.87–8.28)	2.76 (0.89–8.53)
	*P* for trend		0.333	0.228	0.186

Time of smoking					
	None (reference)	18 (12.9)	1	1	1
	≤20 years	10 (14.3)	1.16 (0.50–2.66)	1.88 (0.71–4.98)	1.91 (0.70–5.21)
	>20 years	11 (16.9)	1.42 (0.63–3.20)	0.92 (0.37–2.30)	1.01 (0.39–2.56)
	*P* for trend		0.406	0.999	0.856

Cumulative smoking					
	None (reference)	18 (12.9)	1	1	1
	≤20 pack-years	7 (9.3)	0.69 (0.28–1.74)	0.75 (0.27–2.07)	0.77 (0.27–2.21)
	>20 pack-years	14 (21.9)	1.88 (0.87–4.07)	1.66 (0.69–3.98)	1.79 (0.73–4.39)
	*P* for trend		0.167	0.314	0.255

Variables are included as continuous variables					
	None (reference)		1	1	1
	CPD		1.02 (0.99–1.04)	1.02 (1.00–1.05)	1.02 (1.00–1.05)
	*P*		0.18	0.09	0.051
	None (reference)		1	1	1
	Smoking time (years)		1.01 (0.99–1.03)	1.00 (0.97–1.02)	1.00 (0.97–1.02)
	*P*		0.421	0.678	0.885
	None (reference)		1	1	1
	Cumulative smoking (pack-years)		1.01 (1.00–1.03)	1.01 (0.99–1.03)	1.01 (0.99–1.03)
	*P*		0.083	0.385	0.336

Model A: crude model; Model B: adjusted for age, gender, origin, and occupation; Model C: adjusted for age, gender, origin, occupation, drinking, BMI, WC, and diabetes duration; and CPD: cigarettes per day.

**Table 4:** Effect of smoking exposure on fasting blood-glucose (FBG), hemoglobin A1c (HbA1c), cholesterol (CHO), high-density lipoprotein cholesterol (HDL-C), and systolic pressure.

	Model A					Model B					Model C				
	95% CI					95% CI					95% CI				
CPD	*β*	Lower	Upper	Standard *β*	*P*	*β*	Lower	Upper	Standard *β*	*P*	*β*	Lower	Upper	Standard *β*	*P*
FBG	−0.026	−0.069	0.016	−0.074	0.220	−0.027	−0.067	0.014	−0.075	0.199	−0.026	−0.067	0.015	−0.073	0.209
HbA1c	0.000	−0.023	0.022	−0.002	0.976	0.000	−0.022	0.023	0.001	0.983	−0.001	−0.022	0.021	−0.004	0.951
CHO	−0.013	−0.028	0.002	−0.109	0.093	−0.013	−0.027	0.002	−0.108	0.092	−0.013	−0.028	0.001	−0.113	0.073
HDL-C	−0.006	−0.009	−0.004	−0.323	<0.001	−0.006	−0.008	−0.004	−0.316	<0.001	−0.006	−0.008	−0.004	−0.312	<0.001
Systolic pressure	0.044	−0.265	0.176	0.025	0.693	0.041	−0.255	0.174	0.023	0.711	0.031	−0.241	0.180	0.018	0.775

Time of smoking															
FBG	−0.029	−0.069	0.011	−0.086	0.151	−0.013	−0.053	0.027	−0.039	0.516	−0.016	−0.056	0.024	−0.046	0.440
HbA1c	0.001	−0.019	0.022	0.009	0.889	0.006	−0.015	0.027	0.037	0.585	0.000	−0.021	0.020	−0.002	0.970
CHO	−0.011	−0.025	0.003	−0.103	0.112	−0.006	−0.020	0.008	−0.052	0.427	−0.008	−0.022	0.006	−0.070	0.282
HDL-C	−0.005	−0.007	−0.002	−0.245	<0.001	−0.004	−0.006	−0.002	−0.223	<0.001	−0.004	−0.007	−0.002	−0.231	<0.001
Systolic pressure	−0.006	−0.223	0.211	−0.003	0.958	−0.077	−0.293	0.138	−0.045	0.480	−0.052	−0.264	0.160	−0.030	0.631

Cumulative smoking															
FBG	−0.020	−0.049	0.010	−0.078	0.194	−0.011	−0.040	0.018	−0.046	0.439	−0.011	−0.040	0.018	−0.043	0.467
HbA1c	−0.004	−0.019	0.012	−0.029	0.652	−0.002	−0.017	0.014	−0.013	0.843	−0.002	−0.017	0.013	−0.016	0.796
CHO	−0.009	−0.019	0.002	−0.105	0.105	−0.006	−0.016	0.004	−0.072	0.265	−0.006	−0.016	0.004	−0.077	0.230
HDL-C	−0.002	−0.005	0.000	−0.141	0.058	−0.002	−0.005	0.000	−0.134	0.059	−0.002	−0.005	0.000	−0.128	0.051
Systolic pressure	0.010	−0.144	0.163	0.008	0.901	0.019	−0.170	0.132	0.015	0.807	0.021	−0.169	0.126	0.018	0.776

Model A: crude model; Model B: adjusted for age, sex, occupation, and region; Model C: adjusted for age, sex, occupation, region, drinking, BMI, WC, and duration of diabetes; and CPD: cigarettes per day.

## Data Availability

The datasets used to support this study are not freely available in view of participants' privacy protection.

## Supplementary Materials

Supplementary Materials Figure S1: the conceptual diagram of model 4.
