# Supplementary figures and images for: Smoking Is a Risk Factor of Coronary Heart Disease through HDL-C in Chinese T2DM Patients: A Mediation Analysis
Source: J Healthc Eng. 2020 Jul 28;2020:8876812. doi: 10.1155/2020/8876812 (PMC7407009; doi:10.1155/2020/8876812)

## Slide 1
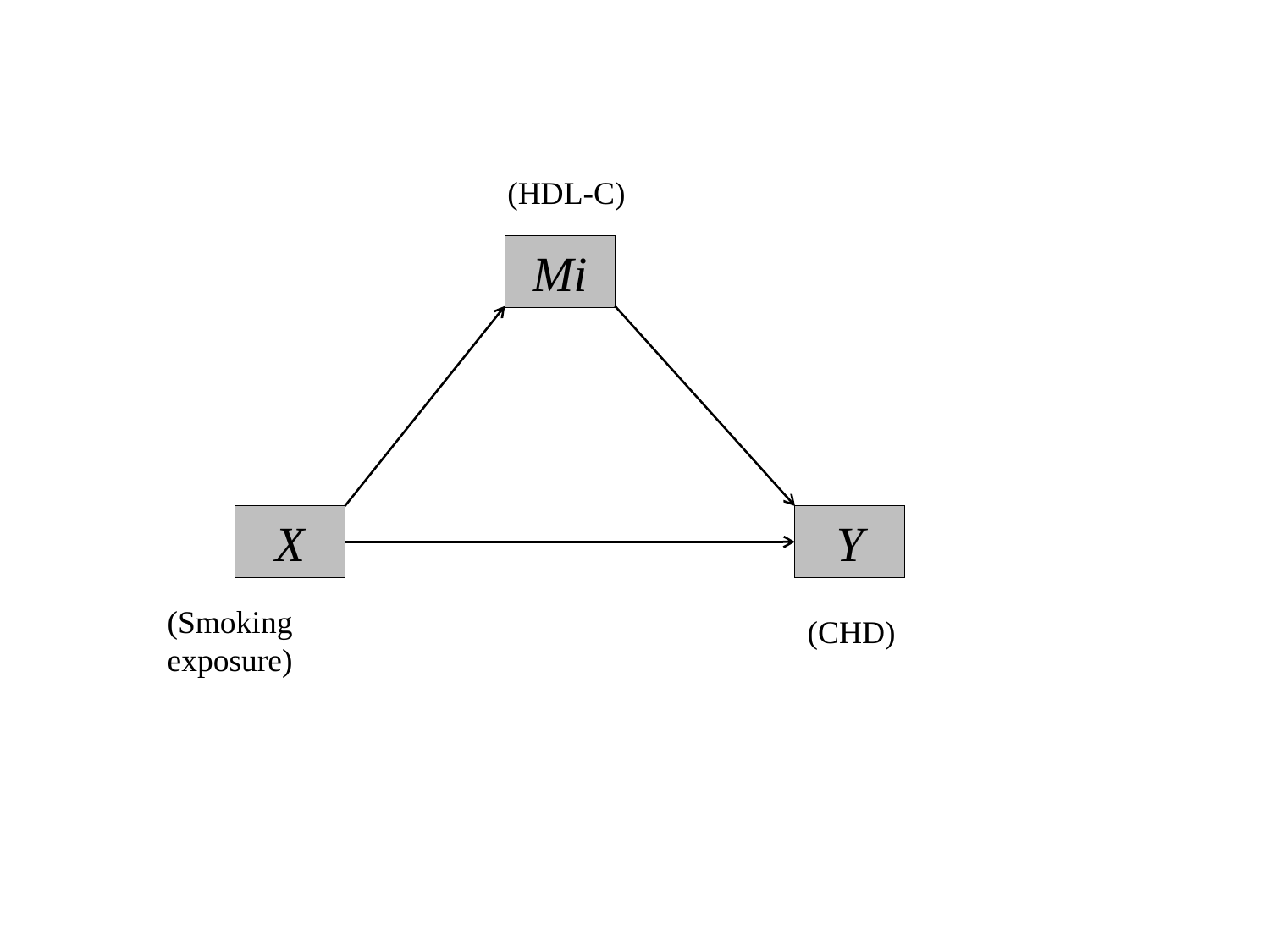

(HDL-C)
Mi
X
Y
(Smoking exposure)
(CHD)

Supplement: Supplementary Materials — Figure S1: the conceptual diagram of model 4. [file 8876812.f1.pptx]
